# Identification of five hub genes as monitoring biomarkers for breast cancer metastasis in silico

**DOI:** 10.1186/s41065-019-0096-6

**Published:** 2019-06-21

**Authors:** Yun Cai, Jie Mei, Zhuang Xiao, Bujie Xu, Xiaozheng Jiang, Yongjie Zhang, Yichao Zhu

**Affiliations:** 1 Department of Physiology, Nanjing Medical University, Nanjing, 211166 China; 2 Department of Bioinformatics, Nanjing Medical University, Nanjing, 211166 China; 3 Department of Human Anatomy, Nanjing Medical University, Nanjing, 211166 China; 4 Key Laboratory for Aging & Diseases of Nanjing Medical University, Nanjing Medical University, Nanjing, 211166 China; 5 State Key Laboratory of Reproductive Medicine, Nanjing Medical University, Nanjing, 211166 China

**Keywords:** Breast cancer, WGCNA, Bioinformatic analysis, Prognosis, Metastasis

## Abstract

**Background:**

Breast cancer is one of the most common endocrine cancers among females worldwide. Distant metastasis of breast cancer is causing an increasing number of breast cancer-related deaths. However, the potential mechanisms of metastasis and candidate biomarkers remain to be further explored.

**Results:**

The gene expression profiles of GSE102484 were downloaded from the Gene Expression Omnibus (GEO) database. Weighted gene co-expression network analysis (WGCNA) was used to screen for the most potent gene modules associated with the metastatic risk of breast cancer, and a total of 12 modules were identified based on the analysis. In the most significant module (R^2^ = 0.68), 21 network hub genes (MM > 0.90) were retained for further analyses. Next, protein-protein interaction (PPI) networks were used to further explore the biomarkers with the most interactions in gene modules. According to the PPI networks, five hub genes (*TPX2, KIF2C, CDCA8, BUB1B*, and *CCNA2*) were identified as key genes associated with breast cancer progression. Furthermore, the prognostic value and differential expression of these genes were validated based on data from The Cancer Genome Atlas (TCGA) and Kaplan-Meier (KM) Plotter. Receiver operating characteristic (ROC) curve analysis revealed that the mRNA expression levels of these five hub genes showed excellent diagnostic value for breast cancer and adjacent tissues. Moreover, these five hub genes were significantly associated with worse distant metastasis-free survival (DMFS) in the patient cohort based on KM Plotter.

**Conclusion:**

Five hub genes (*TPX2*, *KIF2C*, *CDCA8*, *BUB1B*, and *CCNA2*) associated with the risk of distant metastasis were extracted for further research, which might be used as biomarkers to predict distant metastasis of breast cancer.

## Background

Breast cancer is one of the most common tumours threatening women’s health worldwide, and its incidence has shown a rising trend in the past decades. According to the prediction provided by the American Cancer Society (ACS), there will be more than 271,000 new incidences in the United States (US) in 2019 [1]. Current therapeutic approaches for breast cancer mainly focus on comprehensive treatment, including surgery, chemotherapy, radiotherapy, hormone therapy, and targeted therapy [2, 3]. Breast cancer in situ is usually not fatal to patients; however, advanced breast cancer with lymph nodes and/or distant metastasis tends to cause life-threatening outcomes for patients [4]. Although the aetiology and signatures of breast cancer have been preliminarily explored, there is no defined means of predicting the metastasis and recurrence of breast cancer in clinical practice [5], and further exploration of the potential mechanisms of the metastasis and biomarkers to monitor recurrence of breast cancer is urgently needed.

With the continuous development of biological research technologies, especially sequencing technologies and bioinformatic algorithms, massive amounts of genomic information are accumulating exponentially [6, 7]. Along with the successful implementation of numerous large-scale sequencing projects led by governments, biomedical research has been entering the era of “big data” [8–10]. Over the past few years, bioinformatics researchers have developed a series of analysis strategies and data mining algorithms for increasing the amount of transcriptomic data [11–13].

Weighted gene co-expression network analysis (WGCNA) is a systematic biological strategy for evaluating gene association patterns among different samples [11]. It can be applied to reveal highly correlative gene sets and explore potential biomarker genes or therapeutic targets according to the internal connectivity of gene clusters and the associations between gene clusters and phenotypes. By constructing a gene co-expression network and identifying related gene clusters, the correlation between gene modules and phenotypes can be calculated based on phenotypic information, and the most relevant gene modules can be found. Numerous potential biomarkers have been identified to date based on WGCNA for sequencing data [14–16]. For example, Tang et al. identified five genes as prognostic biomarkers for breast cancer, and Qiu et al. revealed several genes associated with the development of breast cancer for further basic and clinical research [14, 17]. However, there is currently no defined means of predicting the metastasis risk of breast cancer in clinical practice. Thus, we aim to further explore potential metastatic predictors via WGCNA.

GSE102484, a microarray containing 683 breast cancer samples, was submitted by Cheng et al. from Taiwan in 2017 [18]. Cheng et al. validated the prognostic value of an 18-gene classifier that predicted locoregional recurrence and metastasis risk in patients after mastectomy based on the sequencing data and clinical information of GSE102484 [18, 19]. Given the complete follow-up information and the defined risk scoring system of recurrence and metastasis for this microarray data, we re-evaluated these data in this study and identified five candidate biomarkers associated with the risk of metastasis through WGCNA and protein-protein interaction (PPI) networks. We further validated the differential expression and prognostic value of these five genes with another open database. Overall, we identified five genes (*TPX2*, *KIF2C*, *CDCA8*, *BUB1B*, and *CCNA2*) associated with distant metastasis, indicating these genes as potential biomarkers for assessing the risk of breast cancer recurrence and distant metastasis.

## Materials and methods

### Acquisition of microarray data

**Fig. 1:**
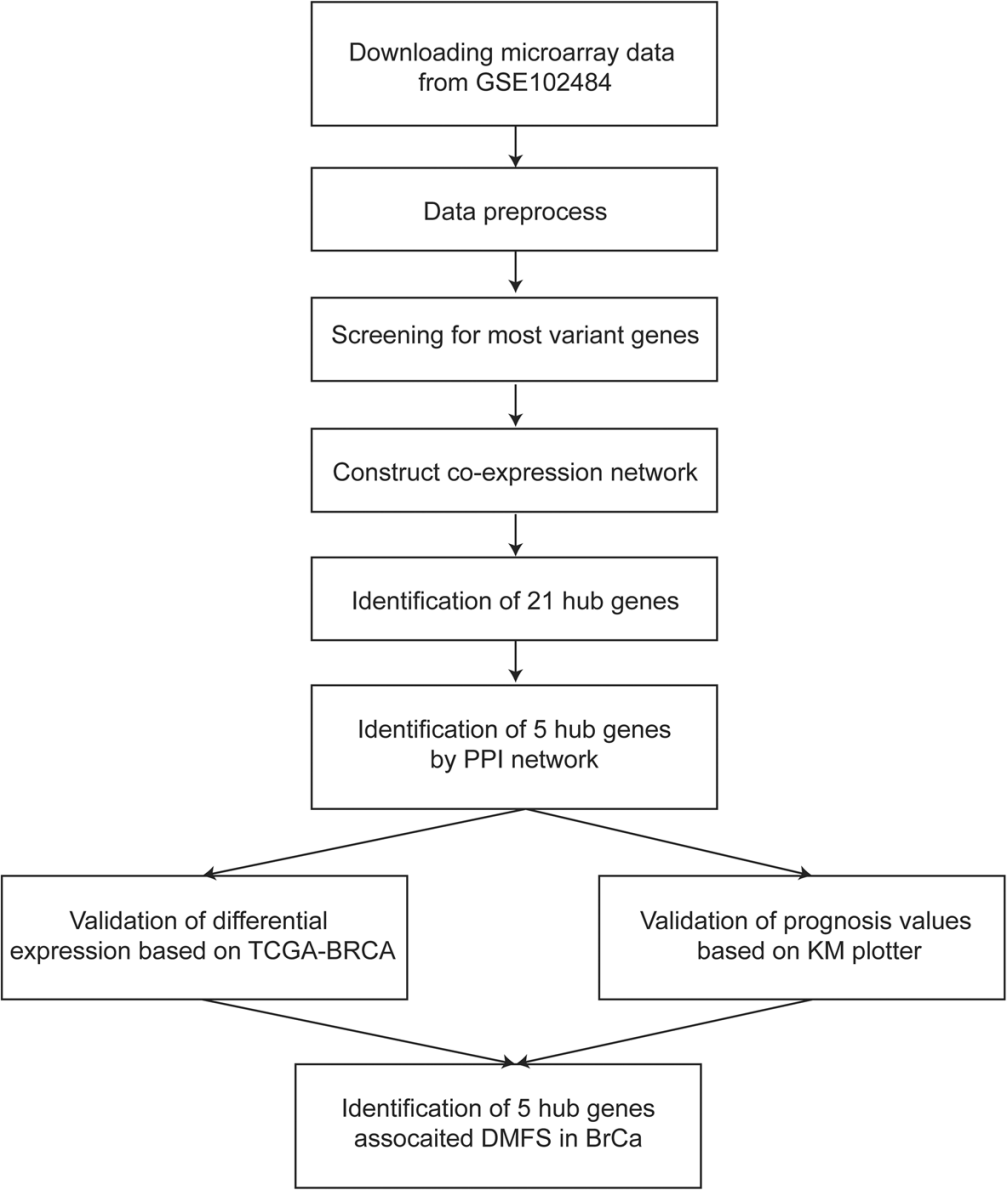
Flow chart of data preparation, processing, analysis, and validation. The gene expression profiles of GSE102484 were downloaded from the GEO database. WGCNA and PPI networks were further used to investigate potential biomarkers associated with the clinical stages and risk of distant metastasis. In addition, the prognostic value and differential expression of hub genes were validated based on data from TCGA-BRCA and KM Plotter

The workflow of our research is illustrated in Fig. 1. The array profiles of GSE102484 (https://www.ncbi.nlm.nih.gov/geo/query/acc.cgi?acc=GSE102484) contributed by Cheng et al. were downloaded from the Gene Expression Omnibus (GEO) database. GSE102484 was an array profile based on the GPL570 platform (Affymetrix Human Genome U133 Plus 2.0 Array) containing 683 breast cancer samples from Taiwan. The robust multi-array average (RMA) algorithm in the affy package within Bioconductor (http://www.bioconductor.org) in R was employed to preprocess the array profiles. After background correction, quantile normalization and probe summarization, the data set with 21,653 genes was further processed. The top 25% most variant genes (MVGs) according to analysis of variance (5413 genes) were selected for further WGCNA.

### Co-expression network construction

After preprocessing the GSE102484 microarray data, the expression profile of these 5413 genes was employed to construct to a gene co-expression network using the WGCNA package in R [11]. The idea of a soft threshold is to continually elementize the elements in the Adjacency Matrix through a weight function. Because the choice of the soft threshold, β, is bound to affect the result of module identification and the relative network of the random average of each node, there is a scale-free network in which a few nodes exhibit a significantly higher degree than the general point, which is a more stable choice, so we need to choose a soft threshold, β, is our gene distribution in line with the scale-free network. To create a network with a nearly scale-free topology, we installed the soft threshold power of β = 4 (scale free R^2^ = 0.88). Adjacency matrices were calculated and transformed into the topological overlap matrix (TOM). The dynamic tree cut algorithm was applied to detect gene modules. Gene significance (GS) was defined as the correlation coefficient between gene expression and module traits. The module eigengene was calculated as a summary profile for each module. Module significance was defined as the correlation coefficient between a module’s eigengene and traits. Module membership (MM) was defined by the correlation coefficient of the module eigengene and gene expression profile. Genes with MM values above 0.90 were considered to be the modules’ representative genes with potential critical functions.

### Identification of hub genes based on a PPI network

After WGCNA of the microarray data, we screened 21 hub genes in the yellow module, which were significantly associated with clinical stage, T stage, and risk of metastasis. We constructed the protein–protein interaction (PPI) network using the STRING database (https://string-db.org/) to load all the hub genes [20]. For all other parameters, the default settings were used. *.tsv format network files were loaded into the plug-in cytoHubba based on Cytoscape software version 3.5.1 (Institute for Systems Biology, Seattle, WA, USA). We defined the top 5 genes with the highest prediction scores calculated by the Stress algorithm as hub genes. We defined these genes with a minimum required interaction score greater than 0.90 as hub genes according to the PPI network, and the network diagram of all PPI hub genes was visualized with Cytoscape (version 3.5.1, Seattle, WA) [12].

### Further authentication of key genes using other open data

To verify the significant values of five hub genes, the clinical files and RNA-seq data for breast cancer were downloaded from The Cancer Genome Atlas (TCGA, https://cancergenome.nih.gov/) database. The mRNA sequencing data were normalized using the edge R package in R software, and we used Student’s *t*-test and the receiver operating characteristic (ROC) curve to examine the differential mRNA levels of these five genes in breast cancer and adjacent tissues. The prognostic impacts of these five genes in breast cancer were analysed using the Kaplan-Meier Plotter (http://kmplot.com/analysis/), an online database containing gene expression profiles and survival data for breast cancer patients [21]. According to the mRNA expression of the genes, the cases in the database were ranked from high expression to low expression, which divided them into two groups; the top 50% were divided into the high expression group, and the bottom 50% belonged to the low expression group. All cohorts were compared with Kaplan-Meier survival plots. The hazard ratio (HR), 95% confidence interval (95%CI), and log rank *P* value were calculated and displayed online.

### Statistical analysis

All statistical analyses were performed using SPSS 25.0 software and R 3.5.1 software. Most statistical comparisons among different groups were analysed with Student’s *t*-test and one-way analysis of variance (ANOVA). The ROC) curve was applied to examine the diagnostic value of differential hub gene mRNA expression for distinguishing tumour tissues from normal tissues. Kaplan-Meier survival plots were generated with survival curves compared by the log-rank test. For all analyses, differences were considered statistically significant if the *P* values were less than 0.05.

## Results

### Weighted co-expression network construction and key module identification

**Fig. 2:**
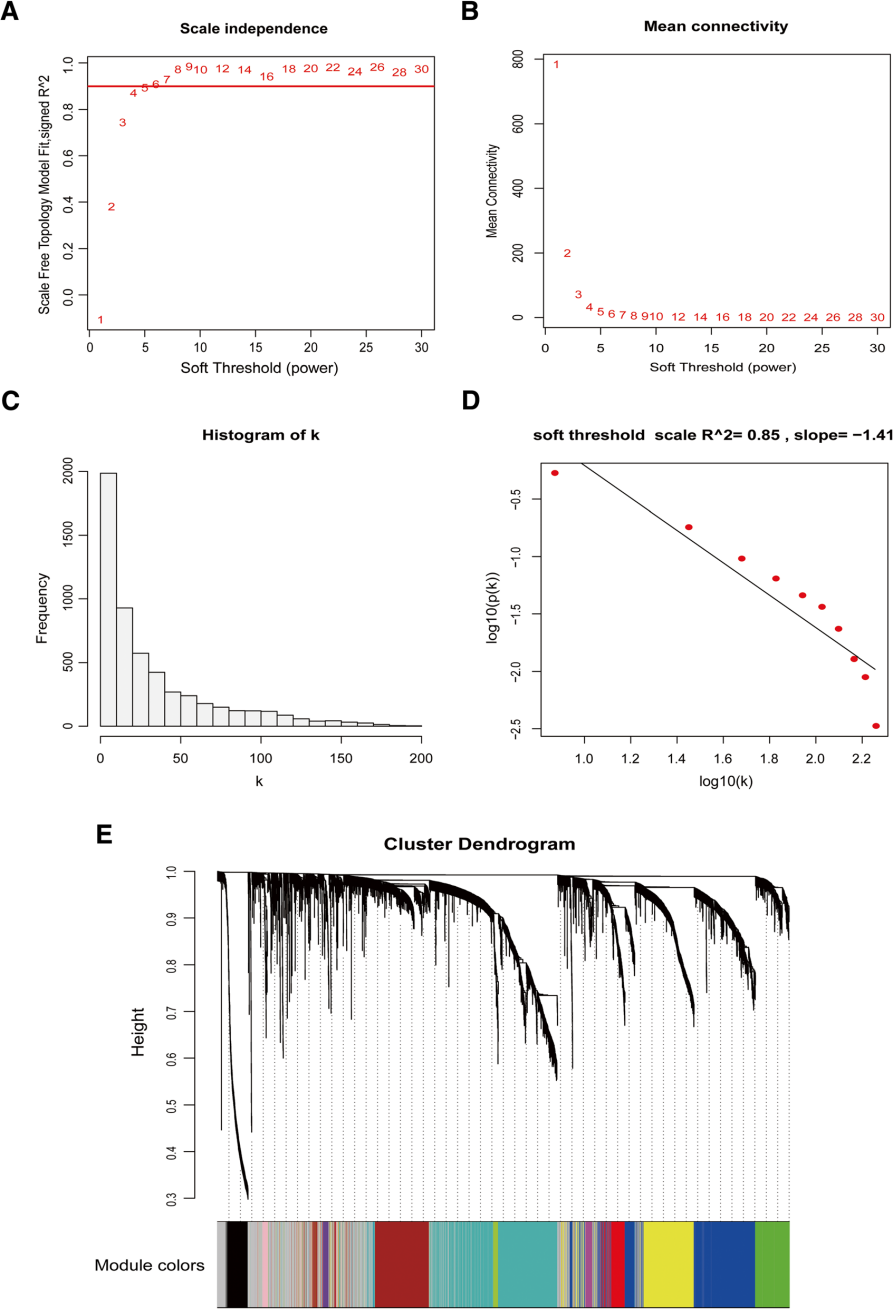
Determination of soft-thresholding power in WGCNA. (**a**) Analysis of the scale-free fitting indices for various soft-thresholding powers (β). (**b**) Mean connectivity analysis of various soft-thresholding powers. (**c**) Histogram of the connection distribution when β = 4. (**d**) Checking the scale-free topology when β = 4. According to Fig. 2C and D, k and p(k) are negatively correlated (correlation coefficient 0.85), indicating that a gene scale-free network can be resumed. (E) Clustering dendrograms of genes based on dissimilarity topological overlap and module colours. As a result, 12 co-expression modules were constructed and are shown in different colours. These modules are arranged from large to small according to the number of genes included

**Fig. 3:**
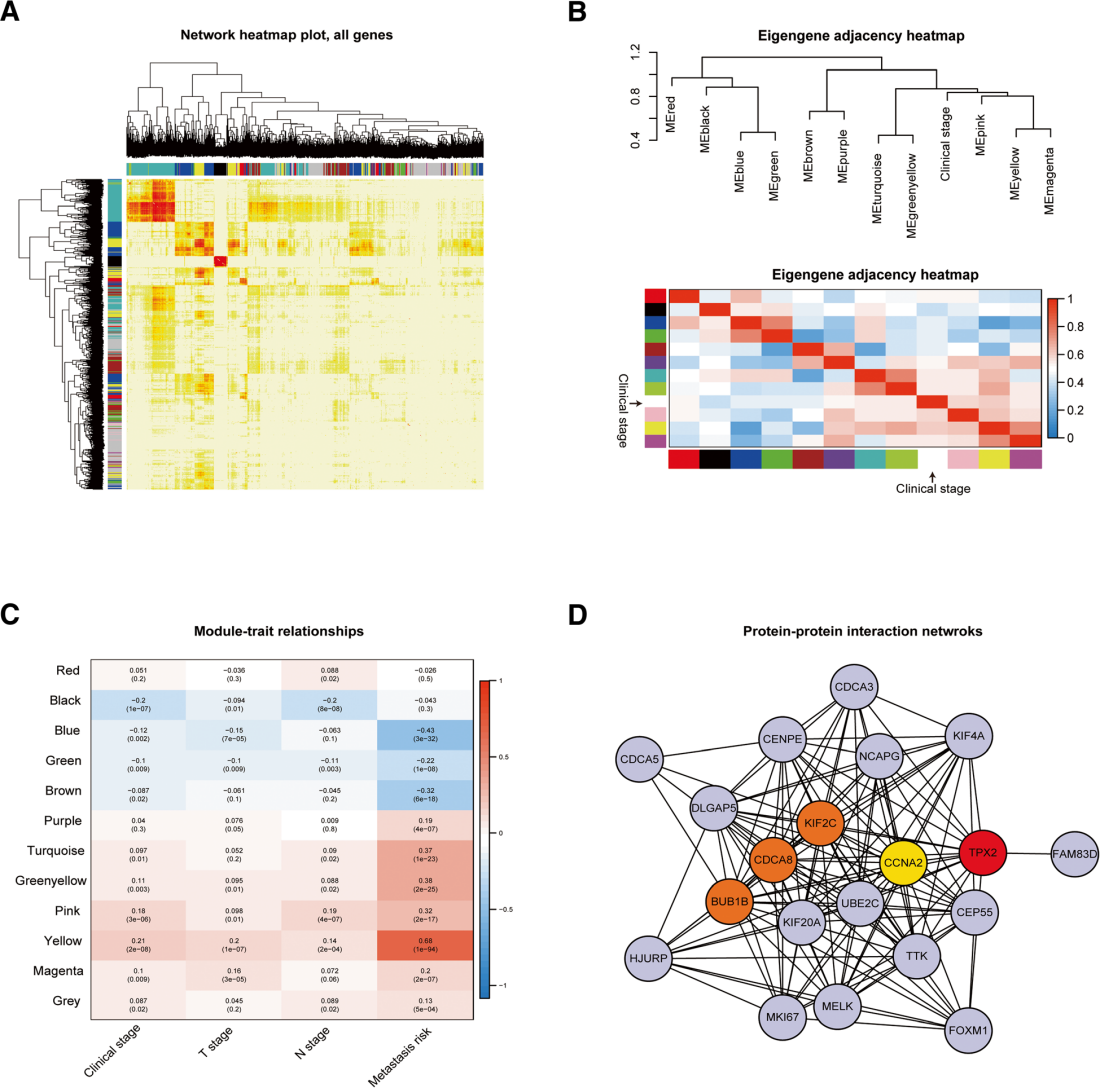
Identification of relevant modules of breast cancer clinical traits and construction of the PPI network. (**a**) Visualization of the gene network with a heatmap. The heatmap depicts the TOM among all genes in the analysis. A light colour represents low overlap, and progressively darker reds indicate higher overlap. Blocks of darker colours along the diagonal are the modules. The gene dendrogram and module assignment are also shown along the left and upper sides. (**b**) The eigengene dendrogram and heatmap identify groups of correlated eigengenes termed meta-modules. As a result, the dendrogram shows that yellow, pink and green-yellow modules are highly related to breast cancer clinical stage. (**c**) Heatmap of the correlation between module eigengenes and clinical traits of breast cancer. The yellow gene module was shown to exhibit the strongest correlation with the clinical stage (clinical stage, T stage, N stage, metastatic risk). (**d**) PPI network of genes in the yellow module for clinical stage. The nodes other than the pale blue nodes represent the hub genes with the highest prediction scores calculated with the stress algorithm

The R package for WGCNA was applied to construct a co-expression network, and 2370 MVGs with similar expression patterns were submitted to modules by cluster analysis. In our research, we selected the power of β = 4 (scale free R^2^ = 0.88) as the soft threshold to ensure a scale-free network (Fig. 2A-D). Then, we extracted twelve modules for further analysis (Fig. 2E). We next visualized the gene network with a heatmap and meta-modules (Fig. 3A, B). The yellow module, which was most significantly associated with clinical stage, T stage, and metastasis risk, was shown to be of notable value in the evaluation of breast cancer progression. Clinical stage (R = 0.21; *P* = 2e− 8), T stage (R = 0.20; *P* = 1e− 7) and metastasis risk (R = 0.68; P = 1e− 94) were all significantly correlated with the yellow module according to module-feature relationship analysis (Fig. 3C). Subsequently, we selected 21 genes in the yellow module with MM values above 0.90 for further analysis, which were considered representative genes exhibiting potential critical functions.

### Identification of hub genes of modules and filtration via the PPI network

In this study, we extracted 21 hub genes with a high correlation with the yellow module (Fig. 3C). Subsequently, we constructed a PPI network using Cytoscape, and we screened these 21 PPI hub genes exhibiting intimate interactions with other genes (Fig. 3D). Based on the stress algorithm, we retained the top five genes (*TPX2*, *KIF2C*, *CDCA8*, *BUB1B*, and *CCNA2*) showing the closest connections with other genes for validation and subsequent investigation (Table 1).

**Table 1:** Five hub genes identified by combined bioinformatic strategies in breast cancer

Gene ID	Ensembl ID	Gene Description
TPX2	ENSG00000088325.15	Targeting protein for *Xenopus* kinesin-like protein 2
KIF2C	ENSG00000142945.12	Kinesin family member 2C
CDCA8	ENSG00000134690.10	Cell division cycle associated 8
BUB1B	ENSG00000156970.12	BUB1 mitotic checkpoint serine/threonine kinase B
CCNA2	ENSG00000145386.9	Cyclin A2

### Validation of hub genes based on TCGA-BRCA data

**Fig. 4:**
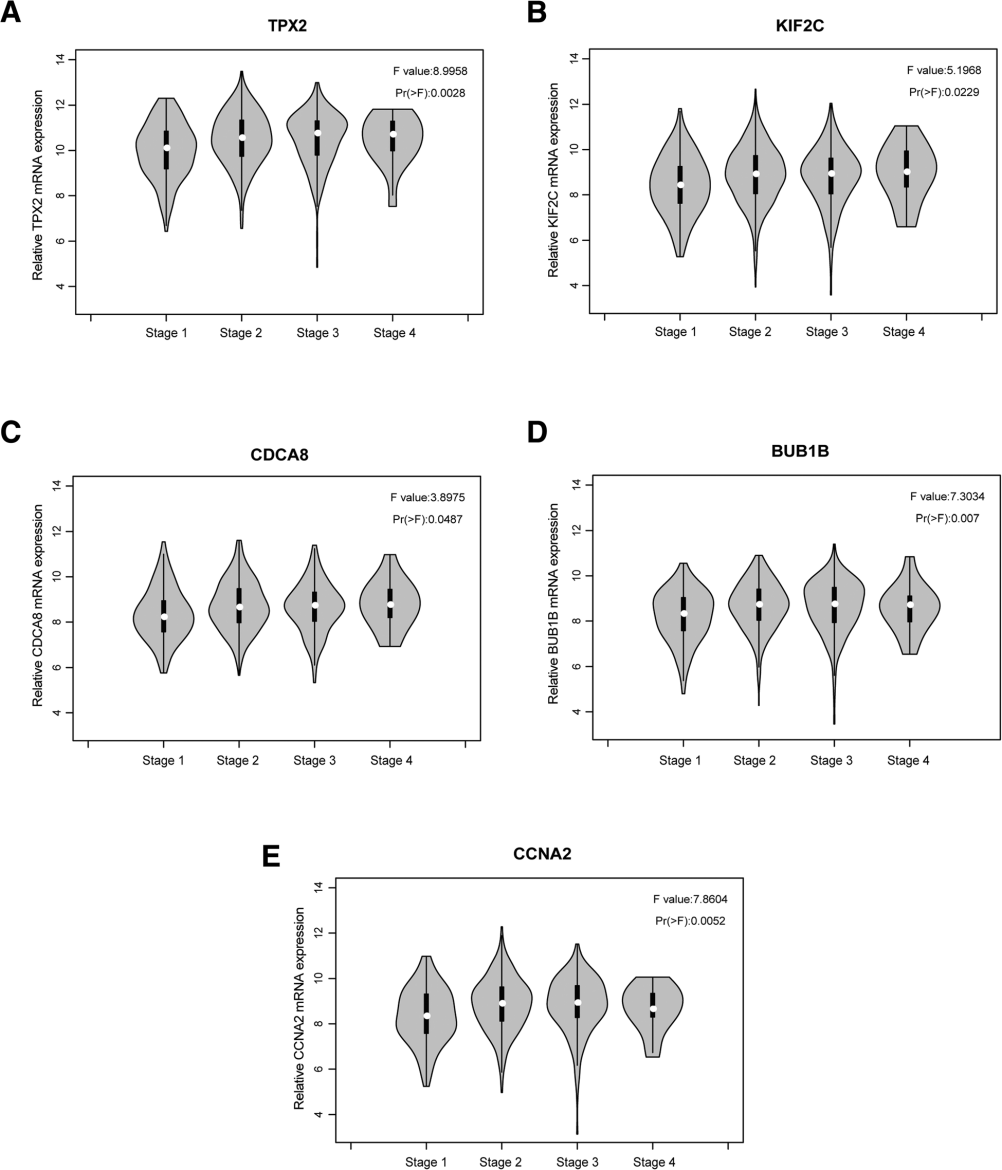
Validation of the differential expression of five hub genes in various clinical stages. (**a**) The correlation of TPX2 expression with clinical stage. (**b**) The correlation of KIF2C expression with clinical stage. (**c**) The correlation of CDCA8 expression with clinical stage. (**d**) The correlation of BUB1B expression with clinical stage. (**e**) The correlation of CCNA2 expression with clinical stage. ANOVA was used to assess the statistical significance of the differences

**Fig. 5:**
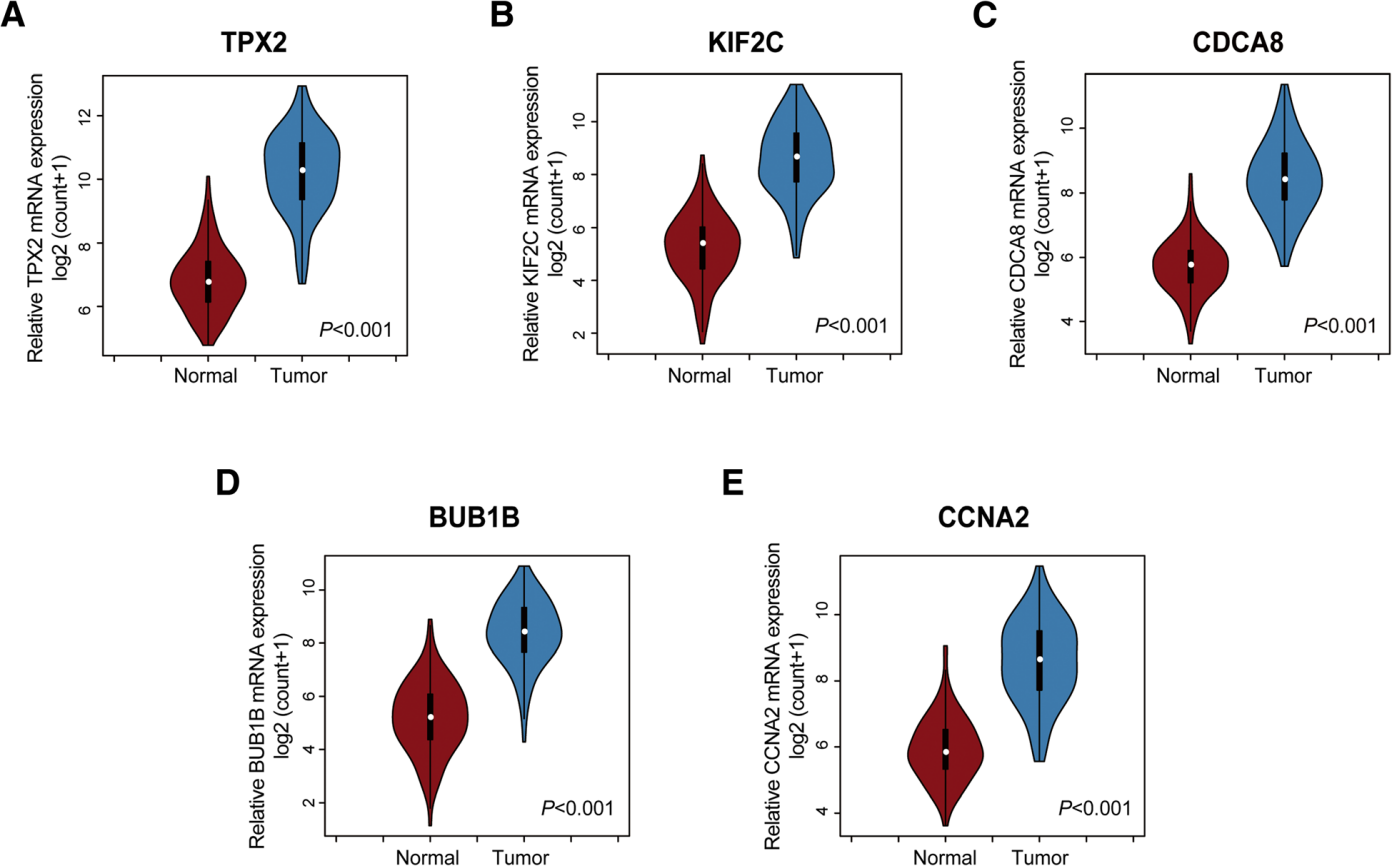
Expression of the five hub genes between normal and breast cancer tissues. The mRNA levels of (A) TPX2, (B) KIF2C, (C) CDCA8, (D) BUB1B, (E) CCNA2, were significantly upregulated in breast cancer tissues compared with adjacent breast tissues. Two-tailed Student’s *t*-tests were used to assess the statistical significance of differences

**Fig. 6:**
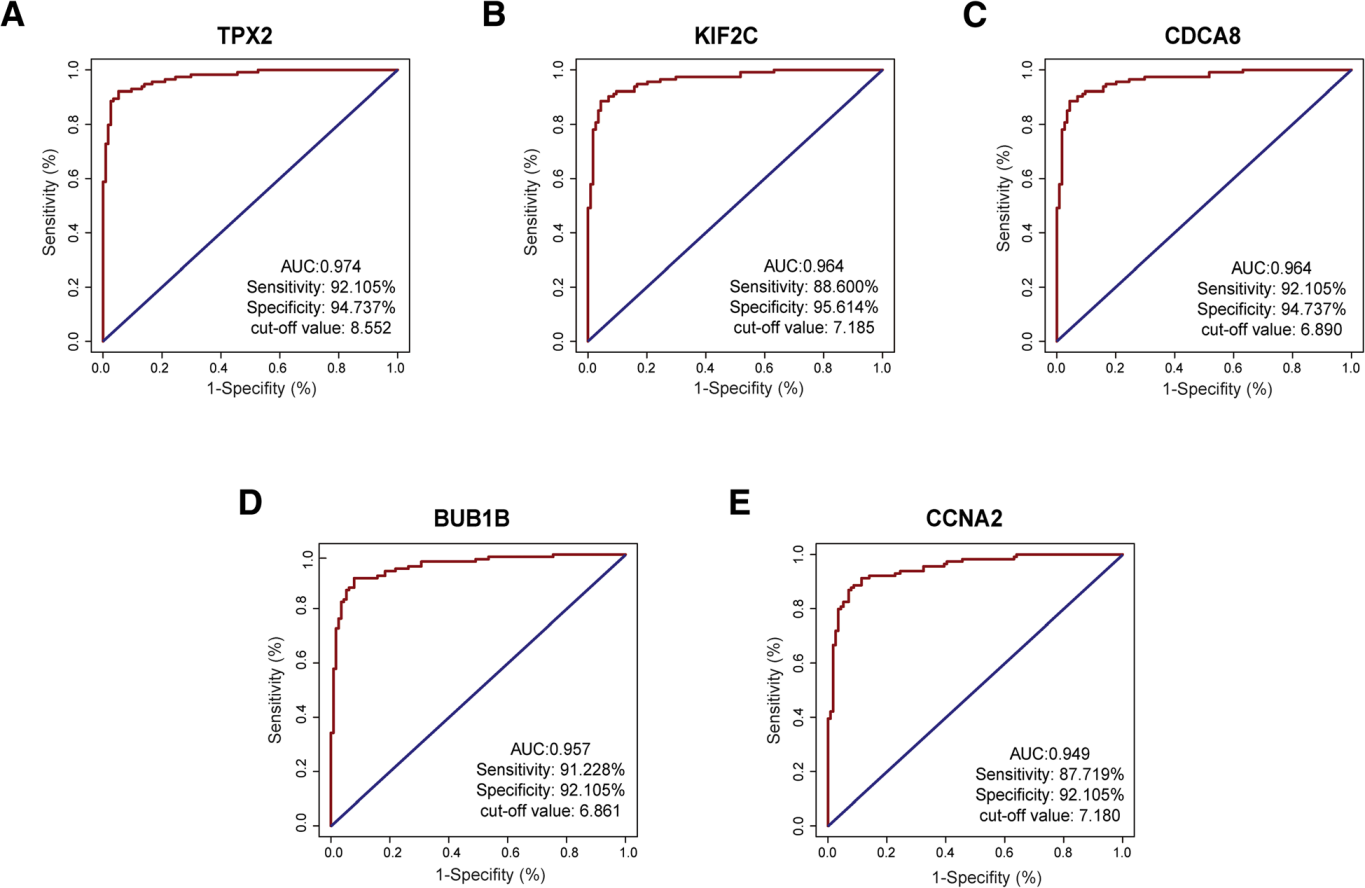
Diagnostic value of the five hub genes in identifying normal and breast cancer tissues. The ROC curve revealed that the mRNA levels of these five genes exhibited excellent diagnostic efficiency for breast cancer and adjacent tissues. (**a**) TPX2, (**b**) KIF2C, (**c**) CDCA8, (**d**) BUB1B, (E) CCNA2

The training dataset (TCGA-BRCA) was applied to validate the correlations between the five hub genes and clinical stages. We compared the expression of each candidate hub gene in breast cancer samples at different clinical stages and found that the five candidate hub genes were closely related to clinical stages (Fig. 4A-4E). In addition, Wang et al. verified a list of cancer/testis genes showing high specificity of testis-specific expression, which included these five hub genes [22]. We speculate that these five genes were upregulated in tumour tissues. We used the TCGA-BRCA dataset to validate our hypothesis, and the results showed that the mRNA level of each candidate hub gene was significantly upregulated in breast cancer tissues compared with paired adjacent breast tissues (Fig. 5A-5E). In addition, ROC analysis revealed that the mRNA levels of these five genes showed excellent diagnostic value for breast cancer and adjacent tissues (Fig. 6A-6E).

### Prognosis value of key genes for DMFS according to KM plotter

**Fig. 7:**
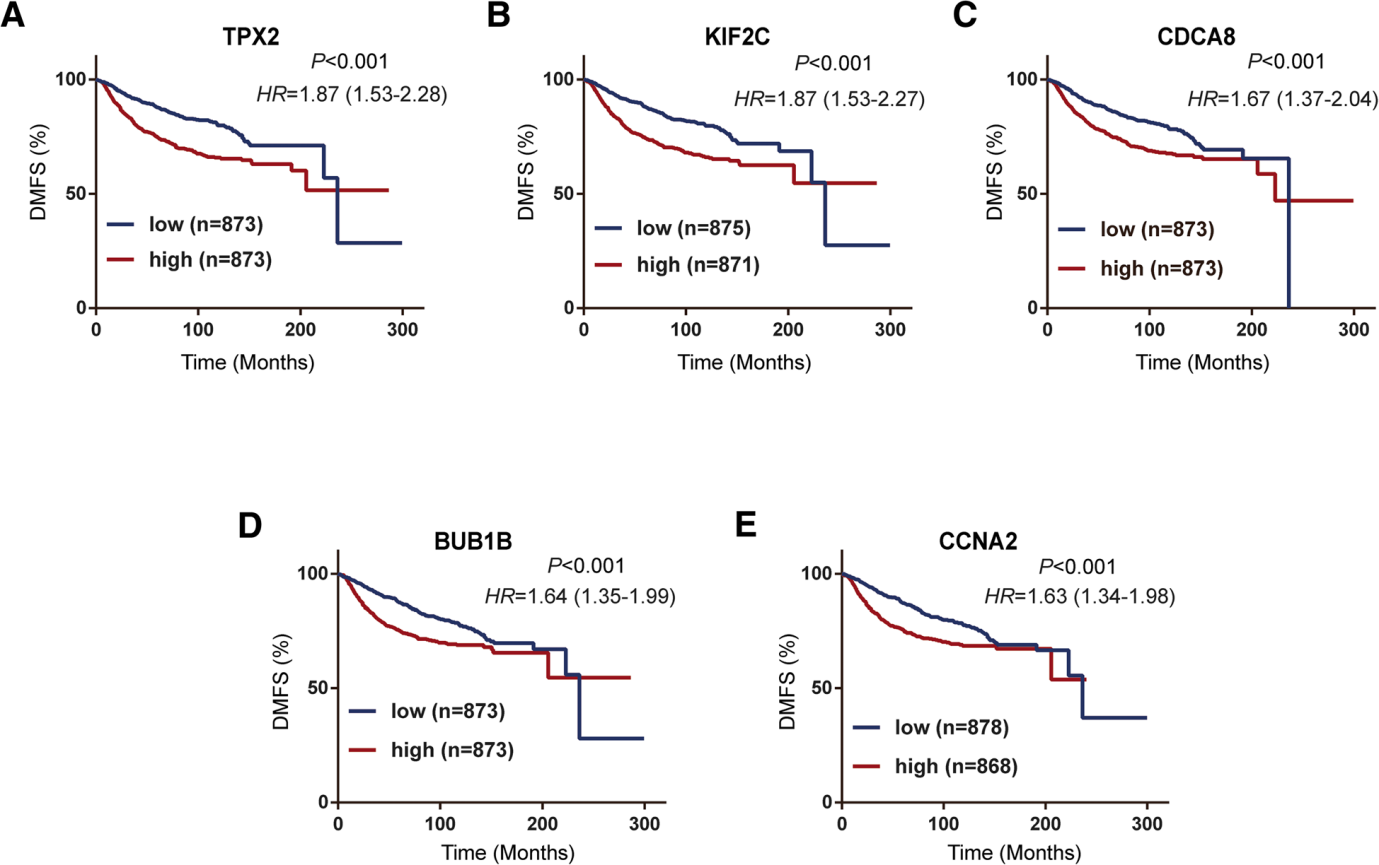
Prognostic value of the five hub genes in breast cancer patients based on KM Plotter. The patients were divided into a high-expression group and a low-expression group according to the median gene expression. (**a**) TPX2 (Affy ID: 210052_s_at). (**b**) KIF2C (Affy ID: 209408_at). (**b**) CDCA8 (Affy ID: 221520_s_at). (**d**) BUB1B (Affy ID: 203755_at). (**es**) CCNA2 (Affy ID: 213226_at)

To validate the association between the expression levels of these five hub genes and metastasis risk in breast cancer, we used survival information from the KM Plotter database to perform survival analysis for the five hub genes. As shown in Fig. 7, high mRNA expression levels of TPX2 (HR = 1.87; 95%CI: 1.53–2.28; *P* < 0.001), KIF2C (HR = 1.87; 95%CI: 1.53–2.27; *P* < 0.001), CDCA8 (HR = 1.67; 95%CI: 1.37–2.04; *P* < 0.001), BUB1B (HR = 1.64; 95%CI: 1.35–1.99; *P* < 0.001), and CCNA2 (HR = 1.63; 95%CI: 1.34–1.98; *P* < 0.001) were significantly associated with worse distant metastasis-free survival (DMFS). Overall, these findings validated the prognostic value and the relationships between the five genes and the metastasis of breast cancer.

## Discussion

Breast cancer is one of the most common cancers that poses a threat to women’s life worldwide. Advanced breast cancer can spread to the whole body through blood vessels and lymphatics and directly cause cancer-related death. Although advanced therapies have achieved promising performance in controlling breast cancer with no metastasis, there are rarely available strategies to control advanced breast cancer, and even precise methods that can prevent the recurrence and metastasis of breast cancer are quite scarce [23, 24]. Therefore, it is urgent to explore potential mechanisms of metastasis and candidate biomarkers for monitoring the metastasis of breast cancer. Although Cheng et al. developed an 18-gene classifier for predicting local/regional recurrence after mastectomy and estimating distant metastasis risk after mastectomy, the complicated scoring system is difficult to widely replicate and apply clinically. Thus, in this study, we re-evaluated microarray data from GSE102484 through WGCNA and PPI analysis and finally identified five genes that exhibited the highest correlation with metastasis risk.

Co-expression analysis is an efficient strategy for gene/disease prediction analysis in large-scale datasets. In the current study, we employed WGCNA to construct a gene co-expression network, to measure the relationships between genes and modules and to investigate the relationships between modules and clinical traits. In the analysis of the top 25% most variant genes (5413 genes), the yellow module was found to show the closest correlation with the risk of metastasis as well as tumour stage, and 21 genes with high connectivity were screened from the module. We next constructed a PPI network and finally identified five (*TPX2*, *KIF2C*, *CDCA8*, *BUB1B*, and *CCNA2*) PPI hub genes that significantly interacted with other genes.

Targeting protein for Xenopus kinesin-like protein 2 (TPX2), which is considered to be a microtubule-associated protein, can participate in the activation of protein kinase activity, regulation of mitotic spindle tissue, apoptotic processes, mitosis, cell division and cell proliferation [25, 26]. Previous studies have shown that TPX2 is overexpressed in tumour tissues and promotes the tumorigenesis of multiple cancers [27–29]. In breast cancer cells, TPX2 promotes cancer cell invasion and migration via regulating MMP2 and MMP9 expression [30]. Additionally, silencing TPX2 can suppress cell proliferation and promote apoptosis by inhibiting PI3K/Akt signalling and activating P53 signalling [31].

Kinesin family member 2C (KIF2C) has been reported to be involved in mitosis and the cell cycle [32]. The encoded protein functions as a microtubule-dependent molecular motor that can polymerize microtubules at the plus end, thus promoting mitotic chromosome segregation. In addition, KIF2C is essential for inducing the deformation of microtubule structures and impairing cell motility [33]. In lung adenocarcinoma, KIF2C is highly expressed and is associated with the recurrence and stage of lung adenocarcinoma [34, 35]. In addition, the expression level of KIF2C mRNA has been identified as a potential independent biomarker for assessing glioma patient prognosis [36]. Accordingly, KIF2C has been reported to be a promising therapeutic target in invasive ductal carcinoma of breast cancer based on PPI network analysis [37].

Cell division cycle-associated 8 (CDCA8) is a gene encoding a component of the chromosomal passenger complex, which functions as a crucial regulator of mitosis and cell division. This protein is required for chromatin-induced microtubule stabilization and spindle formation. Overexpression of CDCA8 in tumour tissues is observed in multiple cancers, including melanoma, bladder cancer, and breast cancer [38–40]. In addition, Dai et al. revealed that the activation of CDCA8 was upregulated by the transcription factor NF-Y in multiple cancer cells [41]. These results indicate that CDCA8 might be a novel target for reducing cell division and proliferation in multiple cancers.

BUB1 mitotic checkpoint serine/threonine kinase B (BUB1B) encodes a kinase participating in the spindle checkpoint; this protein plays a critical role in the inhibition of the anaphase-promoting complex/cyclosome (APC/C) and functions in delaying the onset of anaphase and ensuring proper chromosome segregation [42]. The oncogene role of BUB1B has been observed in cancers such as prostate cancer, glioblastoma and gastric cancer [43–45]. BUB1B is overexpressed in breast cancer, and the level of BUB1B mRNA is significantly correlated with intrachromosomal instability [46]. In addition, BUB1B is preferentially expressed in high-grade breast cancer, and its expression level exhibits significant associations with long-term survival [47].

Cyclin A2 (CCNA2), a protein encoded by the corresponding gene, belongs to the highly conserved cyclin family and functions as a regulator of CDK kinases. This protein binds and activates CDC2 or CDK2 kinases and. Thus. promotes the cell cycle G1/S and G2/M transitions [48]. In breast cancer, NEK5-dependent CCNA2 overexpression promotes the proliferation of tumour cells [49]. Moreover, CCNA2 had significant predictive value for the prognosis of ER+ breast cancer patients as well as tamoxifen resistance [50].

In this research, we further assessed the differential expression of five hub genes (*TPX2*, *KIF2C*, *CDCA8*, *BUB1B*, and *CCNA2*) in various clinical stages, and the results showed that their expression was significantly increased in advanced tumour stages. In addition, the expression levels of the five genes were significantly upregulated in tumour tissues compared with normal tissues. ROC analysis revealed that the mRNA levels of the five genes showed excellent diagnostic value for breast cancer and adjacent tissues. To further assess the association between the expression of these hub genes and the risk of breast cancer metastasis, we employed KM Plotter to evaluate the prognostic value of these genes in DMFS. The primary purpose of KM Plotter is to evaluate potential biomarkers in a meta-analysis-based manner, and many prognostic biomarkers have been identified based on this platform [51–53]. Five hub genes, including *TPX2*, *KIF2C*, *CDCA8*, *BUB1B*, and *CCNA2*, were validated to be notably associated with the shorter DMFS of breast cancer in the patient cohort based on KM Plotter.

## Conclusion

Overall, our results provide valuable indications for biomarker research on breast cancer metastasis. We predicted key metastasis-associated genes based on WGCNA and PPI analysis and validated the primary findings using data from TCGA and KM Plotter. Finally, five hub genes (*TPX2*, *KIF2C*, *CDCA8*, *BUB1B*, and *CCNA2*) were identified for further research, which might be used as promising biomarkers to evaluate the distant metastasis of breast cancer.

## Abbreviations

ANOVA: One-way analysis of variance
BUB1B: BUB1 mitotic checkpoint serine/threonine kinase B
CCNA2: Cyclin A2
CDCA8: Cell division cycle associated 8
DMFS: Distant-metastasis-free survival
GEO: Gene Expression Omnibus
GS: Gene significance
KIF2C: Kinesin family member 2C
KM Plotter: Kaplan-Meier Plotter.
MM: Module membership
MVGs: Most variant genes
PPI: Protein-protein interaction
ROC: Receiver operating characteristic
TCGA: The Cancer Genome Atlas
TOM: Topological overlap matrix
TPX2: Targeting protein for Xenopus kinesin-like protein 2
WGCNA: Weighted gene co-expression network analysis
