# Correction: Identification of five hub genes as monitoring biomarkers for breast cancer metastasis in silico

**DOI:** 10.1186/s41065-023-00300-0

**Published:** 2024-01-19

**Authors:** Yun Cai, Jie Mei, Zhuang Xiao, Bujie Xu, Xiaozheng Jiang, Yongjie Zhang, Yichao Zhu

**Affiliations:** 1 Department of Physiology, Nanjing Medical University, Nanjing, 211166 China; 2 Department of Bioinformatics, Nanjing Medical University, Nanjing, 211166 China; 3 Department of Human Anatomy, Nanjing Medical University, Nanjing, 211166 China; 4 Key Laboratory for Aging & Diseases of Nanjing Medical University, Nanjing Medical University, Nanjing, 211166 China; 5 State Key Laboratory of Reproductive Medicine, Nanjing Medical University, Nanjing, 211166 China

**Correction: Hereditas 156, 20 (2019).**

10.1186/s41065-019-0096-6.

Following publication of the original article [1], the author reported that Fig. 6B and 6C were repeated. The correct Figure is included here and the original article has been updated.

**Fig. 6:**
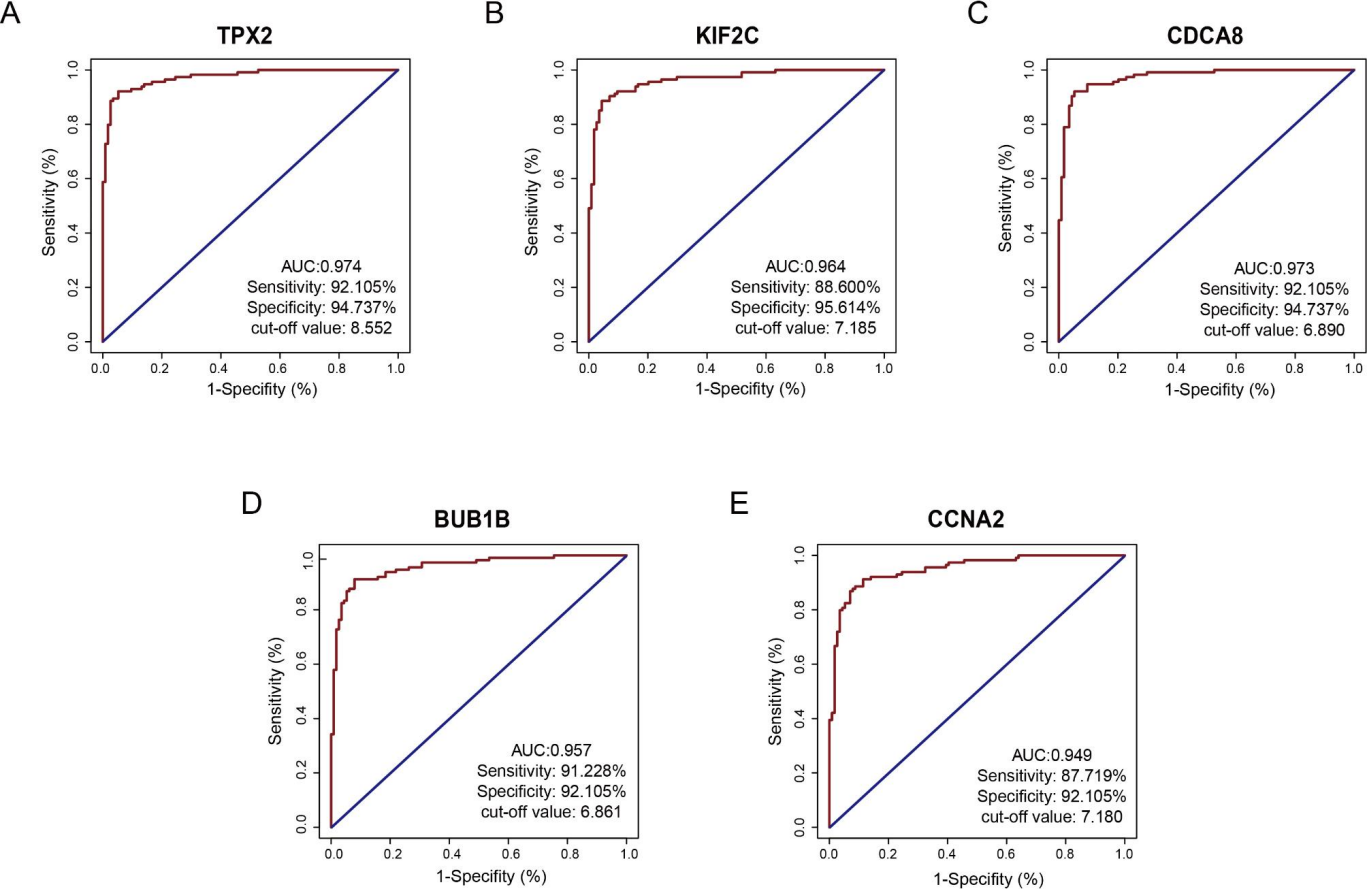
Diagnostic value of the five hub genes in identifying normal and breast cancer tissues. The ROC curve revealed that the mRNA levels of these five genes exhibited excellent diagnostic efficiency for breast cancer and adjacent tissues. **(a)** TPX2, **(b)** KIF2C, **(c)** CDCA8, **(d)** BUB1B, **(E)** CCNA2
